# Efficacy of exercise on balance, fear of falling, and risk of falls in patients with diabetic peripheral neuropathy: a systematic review and meta-analysis

**DOI:** 10.20945/2359-3997000000337

**Published:** 2021-02-25

**Authors:** Renata Aparecida de Oliveira Lima, Gabriela Andrade Piemonte, Célia Regina Nogueira, Vania dos Santos Nunes-Nogueira

**Affiliations:** 1 Universidade do Oeste Paulista Presidente Prudente SP Brasil Fisioterapia pela Universidade do Oeste Paulista, Presidente Prudente, SP, Brasil; 2 Universidade Estadual Paulista Faculdade de Medicina Departamento de Clínica Médica Botucatu SP Brasil Departamento de Clínica Médica, Universidade Estadual Paulista (Unesp), Faculdade de Medicina, Botucatu, SP, Brasil

**Keywords:** Diabetic peripheral neuropathy, exercise, balance, falls, systematic review

## Abstract

Diabetic peripheral neuropathy (DPN) is the most common complication of diabetes mellitus. Our objective was to evaluate the efficacy of exercise interventions in DPN patients from randomized controlled trials. The primary outcomes were the risk of falls, fear of falling, balance and quality of life. Two reviewers independently selected studies from Embase, Medline, LILACS, CENTRAL, and PEDro. They assessed the risk of bias and extracted data from the trials. The relative risk (RR) and the differences between means (MD) were calculated with a 95% confidence interval (CI) and used as the effect size. We used a random-effects model to pool results across studies, and the Grading of Recommendations Assessment, Development, and Evaluation system to evaluate the certainty of evidence. Eight trials were included. No clear effect was observed in the risk of falls (RR: 0.93, 95% CI: 0.41 to 2.09, 79 participants, 1 trial, low-certainty evidence). Regarding fear of falling, using the Falls Efficacy Scale, a small difference in favor of the intervention was observed (MD: −2.42, 95%, CI: −4.7 to −0.15, 3 trials, 185 participants, low-certainty evidence). The meta-analysis of balance using the unipedal stance test showed a small difference in favor of the intervention. One study evaluated quality of life, and in the mental score there was a MD in favor of the intervention. In DPN patients, a combination of gait, balance, and functional training improved balance, the fear of falling, quality of life in the mental score, but not the risk of falls.

## INTRODUCTION

Diabetic peripheral neuropathy (DPN) is the most common complication of type 1 and type 2 diabetes, and cross-sectional studies from the United States and Europe report its prevalence to range from 6% to 51%, depending on the population studied (1–4). The predominant form of DPN is sensory neuropathy with autonomic nervous system involvement (5). DPN is the leading cause of development of diabetic foot ulceration that is the main cause of non-traumatic amputations of the lower limb in high-income countries (5,6). Additionally, patients with DPN can also present with an intrinsic foot muscle dysfunction that may lead to gait abnormalities, compromising balance during daily activities and increasing the risk of falls (7). A population-based survey of African Americans reported that diabetic patients aged 70 years and older had a 2.5-fold increase in falls compared with non-diabetic individuals (8). In a cross-sectional study, using multivariate regression analyzes, the authors showed that age, severity of diabetic neuropathy and depression symptoms are independent predictors of the risk of falls in patients with type 2 diabetes (7). Conversely, falls among older adults are associated with pelvic and hip injuries, avoidance of activities, increased hospitalization leading to substantial economic costs, and mortality (9–11).

Therefore, exercise for improving balance and strengthening the lower extremities has been a part of the non-pharmacological management of DPN. For older populations, these exercises such as resistance, balance, endurance, and coordination training, have already demonstrated beneficial effects on functional parameters (12). This multi-component exercise intervention as well as group and home-based exercise programs and Tai Chi are the best strategies for physically frail older adults (12). This is because, in addition to preventing falls, they stimulate several components of physical health such as strength, cardiorespiratory fitness, and balance (12,13).

In a controlled randomized clinical trial (RCT), an exercise program to improve balance and trunk proprioception in older adults with diabetic neuropathies showed significant improvements in both static and dynamic balance as well as trunk proprioception (14). In a non-RCT, Tai Chi improved glucose control, balance, neuropathic symptoms, and quality of life in DPN patients (15).

A systematic review evaluated the effect of diverse physical rehabilitative interventions on static postural control in DPN. The authors compared exercise programs aimed at improving both static and dynamic balance with standard or conventional forms of physical therapy care. The evaluated outcome was postural control assessment. They concluded that interventions such as balance training, treadmill and strengthening exercises, and whole-body vibration showed improvement in static postural control in patients; however, they did not evaluate either the risk of falls or the fear of falling (16). As they are important outcomes of patient's point of view, and some RCTs have evaluated them (17,18), this review aimed to evaluate the efficacy of exercises composed of strength, endurance, and balance training for the improvement of balance, risk of falls, and the fear of falling in DPN patients.

## METHODS

A systematic review was conducted following the Cochrane Handbook for Systematic Reviews of Interventions (19) and reported according to the Preferred Reporting Items for Systematic Reviews and Meta-Analyses (20). Its protocol was registered in the International Prospective Register of Systematic Reviews under CRD42018087651.

### Eligibility criteria

#### Patients

We included RCTs in which individuals, regardless of gender, aged > 18 years, had a diagnosis of diabetes mellitus and a diagnosis of DPN by one of the following tests: a scoring system for the lower extremity sensations, a neurophysiological study involving electromyography, the vibration sensitivity test using a 128-Hz tuning fork, the tactile sensitivity test (that can be evaluated using the Semmes–Weinstein 5.07 monofilament), or the thermal discrimination test.

#### Intervention

The intervention group comprised patients who participated in an exercise program to improve balance and strength of the lower extremities. Thus, we considered those studies that had all types and combinations of exercises i.e., resistance and non-resistance, aerobic and non-aerobic exercises, as well as Tai Chi.

#### Control

The control group included patients who did not participate in any kind of exercise program.

#### Outcomes

The primary outcomes were the risk of falls, balance as measured using a balance test, such as the Performance-Oriented Mobility Assessment, the Functional Reach Test, Timed Up and Go (TUG), the Berg Balance Scale (BBS), stabilometry or the unipedal stance; the fear of falling measured using the Falls Efficacy Scale (FES) or Activities-specific Balance Confidence Scale (ABC); and the quality of life. The secondary outcomes included the lower extremity neuropathy symptoms; some level of neurological recuperation validated using either the neurophysiological study, electromyography, the vibration sensitivity test, the tactile sensitivity test or the thermal discrimination test; weight loss observed using the body mass index and the waist and/or waist hip ratio; glycemic control (as measured by the fasting blood sugar and glycated hemoglobin), blood pressure control, and adverse events (e.g. hypoglycemia or any other negative event because of exercise).

#### Exclusion criteria

We excluded non- and quasi-RCT, studies with an active comparator, and studies that included patients with other causes of polyneuropathy such as alcoholism, decompensated hypothyroidism, dysproteinemias, anemia, use of potentially neurotoxic drugs, or spinal cord compression.

### Identification of studies

#### Electronic databases

General research strategies were applied to the main electronic health databases: Embase (Elsevier, 1980–31/December/2019), MEDLINE (PubMed, 1966–31/December/2019), LILACS (by Virtual Health Library, 1982–31/December/2019), and Registry of Controlled Clinical Studies of the Cochrane Collaboration (CENTRAL,31/December/2019); which are described in detail in the supplementary data. There was no restriction regarding the language or the year of publication.

We also searched the Trip Medical Database, SCOPUS, Web of Science, and PEDro (Physiotherapy Evidence Database) for eligible studies. We also looked for unpublished studies among dissertations, theses and ClinicalTrials.gov website.

EndNote X9 citation management software was used to download references and remove duplicate entries. The initial screening of abstracts and titles was performed using the free web application Rayyan QCRI (21).

### Study selection

Two reviewers (RAOL and VSNN) independently selected the titles and abstracts identified during the literature search. Potentially eligible studies for inclusion in this review were thoroughly analyzed and subsequently assessed in terms of its appropriateness according to the eligibility criteria. Whenever there was a disagreement in either the selection process, data extraction, or the evaluation of the risk bias, a consensus was reached by discussion.

### Data extraction

For the studies selected for inclusion, RAOL and VSNN independently used a standardized extraction form so that all the information (the number of patients, average age, the inclusion and exclusion criteria, the type of diabetes, the type of treatment, presence of other diabetic complications, the nature of intervention and control groups, outcomes analyzed, the monitoring time, and the risk of bias) regarding each study might be computed.

### Assessment of risk of bias in included studies

For each selected RCT, the risk of bias was independently evaluated by RAOL and VSNN according to the criteria described in the Cochrane Reviewers Handbook (19) that considers seven domains: the process of randomization, concealing allocation, blinding of participants and researchers, blinding of outcome assessors, whether the losses were included in the final analysis, selective reporting of the outcomes, and others.

### Synthesis and analysis

Similar outcomes measured in at least two studies were plotted in a meta-analysis using the Review Manager 5.3 software (the Cochrane Community). Continuous data are expressed as means and standard deviations. Differences between means (MD) with 95% confidence intervals (CIs) were used as an estimate of the intervention effect size. We chose the random-effects model as the analytical model for the meta-analysis. Inconsistencies among the study results were verified by visual inspection of the forest plot (e.g. overlap in the CIs of the estimates of the effect size in the individual studies) and using Higgins or I^2^ statistic. I^2^ > 50% indicated a moderate probability of heterogeneity.

### Quality of evidence

The quality of evidence in the estimation of the effect size of the intervention on the primary outcomes was generated in accordance with the Grading of Recommendations, Assessment, Development, and Evaluation (GRADE) Working Group (22).

## RESULTS

### Selection of studies

**Figure 1:**
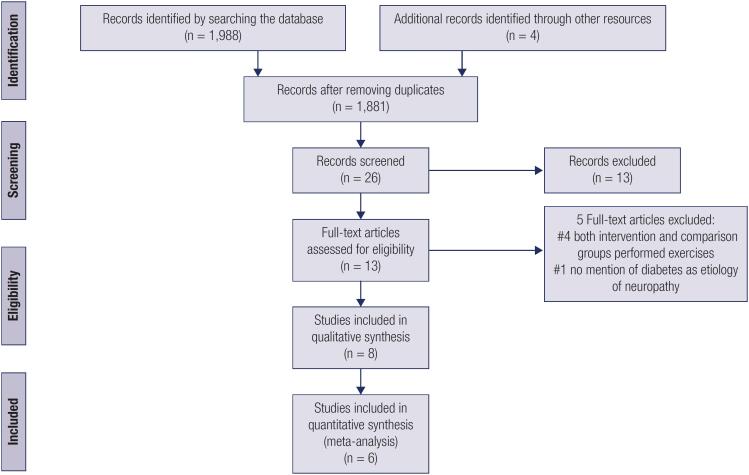
Flowchart for identifying eligible studies.

The search strategies yielded 1,988 references, Figure 1. After removing duplicates, 1,881 studies remained. We selected 13 studies that had a high probability of inclusion in this review, but 5 were excluded in the full text level. Four studies were excluded because both the intervention and control groups were included in an exercise program (23–26), and one study was excluded because it did not mention diabetes as the etiology of the distal symmetric polyneuropathy (27). Eight studies met our eligibility criteria and were included in this review (14,17,18,28–32).

The eight studies analyzed a total of 457 DPN patients. Eligibility criteria, outcomes, country, types of intervention, and the number of participants for the included studies are presented in Table 1. In all these studies, there were no significant differences between the groups at baseline for gender, demographic characteristics, health conditions, measures of balance or lower extremity strength. In five studies, the mean age of the participants was higher than 60 years (14,17,29,31,32); in two studies, the mean age was higher than 50 years (28,30); and in one study, the mean age was 41 and 46 years for the intervention and the control groups, respectively (18). Only Lee 2013 and Grewal 2015 mentioned the glycated hemoglobin (HbA1c) levels of groups at the initial visit (mean of 6.99% vs. 6.93% and mean of 8.1% vs. 8.1% for the intervention and control groups, respectively).

**Table 1:** Characteristics of the included studies, including follow-up, inclusion and exclusion criteria, intervention, control, and outcomes

Study	Country	Nº of Patients Randomized/dropouts	Follow up	Inclusion Criteria	Exclusion Criteria	Intervention	Control	Outcomes
Song *et al.* 2011	South Korea	44/6	8 weeks	DM and DPN	Skeletal muscle impairment, fracture or malformation, severe osteoarthritis, CNS and SV dysfunctions, postural hypotension, mental deficiency, and psychiatric disorders	Physical activities for balance and proprioception of the trunk Education to improve health for DM	Education for DM	1. Static balance: Body sway distance test, unipedal stance test. 2. Dynamic balance: BBS, TUG, FRT, 10-m walking test. 3. Proprioception of the trunk: TREs
Kruse *et al.* 2010	United States	79/5	12 months	T1DM or T2DM and DPN, not having participated in weight lifting exercises for 20 min more than twice a week, loss of sensitivity of at least 1 in 10 points in the feet, loss of vibration sensitivity	Medical contraindication to perform physical activity	Part 1 (1 to 3 months): Physical activities for balance and leg strengthening (8 weeks + 3 weeks more intensively) Program included walking, adapted to the physical capacity of each patient Part 2 (4 to 12 months): Motivational calls for the maintenance of the performance of the activities above, instructions for self-care regarding DM and medical care	Instructions for self-care regarding DM and medical care	1. Static balance: Unipedal stance test. 2. Dynamic balance: BBS, TUG. 3. Ankle muscle strength: dynamometer. 4. Fall: two scales (Falls Efficacy Scale and Function Index Disability Scale) and incidence of one or ≥2 falls throughout the study
Allet *et al.* 2010	Switzerland	71/3	12 weeks	T2DM and DPN (diagnosis based on Rydel-Seiffer tuning fork). No medical contraindication to perform physical activity	Ulcers on the feet, non-diabetic neuropathy, other neurological pathologies that influenced the gait and the incapacity to walk 500 m without support	Twice a week, 60 min, warm-up (5 min), circuit (40 min) that included gait and balance activities, interactive games (10 min) and feedback with suggestions of home exercises (5 min)	Patients have been instructed to maintain their leisure activities, but with no specific orientation	1. Static balance: Postural control by the Biodex Balance System platform (New York, USA) 2. Dynamic balance: Tinetti balance assessment (Performance Oriented Mobility Assessment – POMA), walking as fast and accurately as possible on a 5-meter beam (height: 15 cm and width: 15 cm) 3. Gait: Outdoor gait assessment (Physilog; BioAGM, Lausanne, Switzerland) 4. Fall: Concern of falling was assessed by the Fall Efficacy Scale International (FES-I)
Sartor *et al.* 2014	Brazil	55/16	12 weeks	T1DM or T2DM for at least 7 years, BMI 18.5-29.9 kg/m², DPN (scoring higher than 2 in a maximum of 13 points in the MNSI scale), vibration sensitivity alteration, ability to walk independently, absence of plantar ulceration and amputation	Other neurological and orthopedic disabilities, severe vascular complications, severe retinopathy, or nephropathy	Twice a week, 60 min, exercises to improve the movements of the feet and ankles, strengthen the foot and ankle muscles, increase the ability of walking and foot rollover training	No recommendation regarding physical activity, but medical care was provided continuously	1. Peak pressure on the plantar surface: Peak pressure on the lateral forefoot 2. Foot rollover 3. Kinematic and kinetic variables of the ankle joint 4. Clinical variables (feet physical exam and MNSI)
Dixit *et al.* 2014	India	87/21	8 weeks	T2DM and DPN (with minimum score of 7 in MDNS)	Vitamin B12 deficiency, postural hypotension, foot ulcers, use of walking aids, amputation, PAD, other therapies for DPN and age above 70 years	Aerobic activities according to the AHA guidelines and medical, nutritional, and pedal care	Medical, nutritional, and pedal care	1. Electrophysiological evaluation: Peroneal and sural sensory motor nerves 2. Evaluation of the Michigan Diabetic Neuropathy Score(MDNS)
Lee *et al*. 2013	South Korea	40/4	6 weeks	DM and DPN (medical diagnosis), ≥65 years, two or more falls in the last 12 months, one fall in the TUG or recurrent inexplicable falls	Muscle skeletal disability, MMSE scoring less than 24/30	Training on a vibration platform (Galileo 2000, Novotec Medical GmBH, Germany) (three times a week and 3 min/day) and/or twice a week, 60 min, warm-up (10 min), balance activities (40 min), stretching (10 min)	No participation in physical training	1. Static balance: Body sway distance test, unipedal stance test. 2. Dynamic balance BBS, TUG, FRT. 3. MMII Muscle strengthening: FTSTS. 4. HbA1c
Grewal *et al.* 2015	United States & Qatar	39/5	4 weeks	Ability to walk independently for 20 m and medically diagnosed type 2 diabetes with DPN. DPN was confirmed using the criteria explained in the American Diabetes Association	Presence of cognitive, vestibular, or central neurological dysfunction, musculoskeletal abnormality, active foot ulcers, Charcot's joints, or a history of balance disorder unrelated to DPN	A sensor-based exercise training with real-time visual feedback from the joint motion of the lower extremities to improve the postural stability and activity level + Standard of care	Standard care	1.Fall: Concern of falling was assessed by the Fall Efficacy Scale International (FES-I) 2. Quality of life: Short-form health survey (SF-12) 3. Balance: Postural stability was assessed barefoot in double stance for 30 s with open and closed eyes using a two-link biomechanical model 4. Daily physical activities monitored for 48 h
Kutty *et al.* 2013	India	32/?	6 weeks	Type 2 diabetes, without medical contraindications of engaging in physical activity and with clinically diagnosed diabetic peripheral neuropathy	Concomitant foot ulcers, orthopedic or surgical problems affecting gait variables, nondiabetic neuropathy, and other neurological pathologies	A multisensory exercise program for 30 minutes, 3 times a week over 6 weeks + Usual leisure activities	Usual leisure activities	1. Dynamic balance: TUG, Six-Minute Walk Test

1 T1D: type 1 diabetes mellitus; T2DM: type 2 diabetes mellitus; DPN: diabetic peripheral polyneuropathy; MMII: lower extremity; CNS: central nervous system; AHA: American Heart Association; VS: Vestibular system; TUG test: Timed Up and Go test; TWT: 10-Meter walking test; BMI: Body mass index; ABC Scale: Activities-specific balance; MNSI: Michigan Neuropathy Screening Instrument; MDNS: Michigan Diabetic Neuropathy Score; MMSE: Mini-Mental State Examination; WBV: Whole body vibration; BE: Balance exercise; FTSTS: Five-times-sit-to-stand; HbA1c: Glycated Hemoglobin; ITT: Intention to treat.

Regarding the types of intervention, all studies included applied exercise programs aimed at improving balance and strength of the lower extremities, Table 1. In a nutshell, most patients performed a structured physical activity, which involved gait training by walking, balance training, and lower extremity strength training. Only one study did not present a structured physical activity, rather it mentioned that the supervised physical activity guidelines recommended by the American Heart Association were followed (28).

### Risk of bias among the included studies

**Figure 2:**
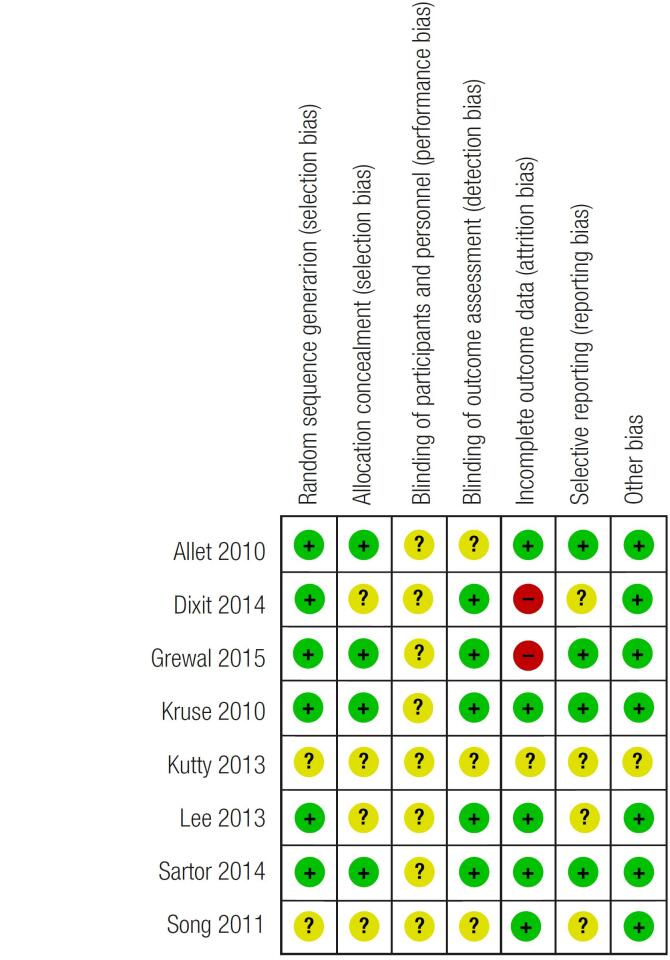
Risk of bias summary: Review of authors’ judgments about each risk of bias item in each included study.

The risks of bias among the included studies are presented in Figure 2. Four studies were classified as having a low risk of selection bias, while the others had an unclear risk. Performance bias was present in all the studies since the participants and the personnel were not blinded to the interventions. Five studies were classified as having a low risk of detection bias. Two studies were evaluated as having a high risk of attrition bias.

### Meta-analysis

The primary outcomes that were plotted in the meta-analysis were the fear of falling that was assessed using the FES, and balance that was assessed using the measures of static and dynamic balance.

**Figure 3:**
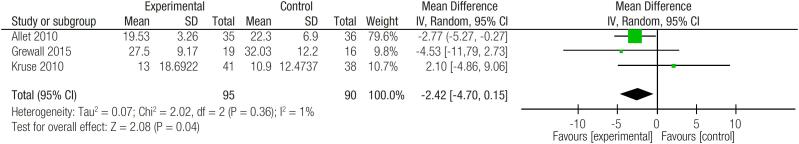
Meta-analysis of the fear of falling, as observed using the Falls Efficacy Scale.

Regarding fear of falling, the meta-analysis showed a small difference in favor of the intervention; however, the quality of evidence was low (MD: −2.42, 95% CI: −4.7 to −0.15, 3 trials, 185 participants, Figure 3, Table 2, supplementary data). This scale is related to daily activities, and a lower score is associated with more confidence in performing certain daily activities.

**Figure 4:**
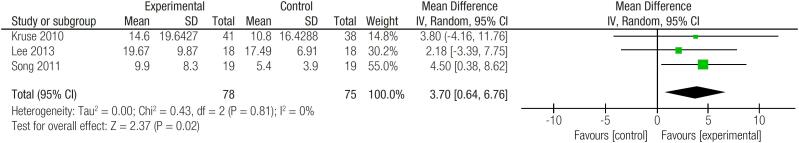
Meta-analysis of the balance, time (in seconds) in the unipedal stance test (left leg – open eyes).

**Figure 5:**
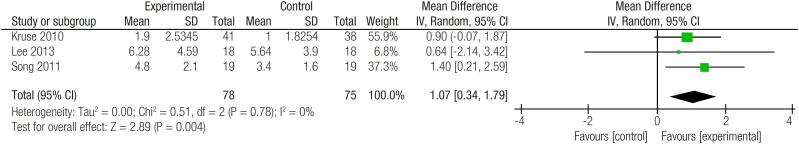
Meta-analysis of the balance, time (in seconds) in the unipedal stance test (left leg – closed eyes).

The meta-analysis of balance was observed using the unipedal stance test (one-leg stance test) with eyes open and closed. It showed a small difference in favor of the intervention, but the quality of evidence was low (MD: 3.7 s, 95% CI: 0.64 to 6.76; MD: 1.07 s, 95% CI: 0.34 to 1.79, 3 trials, 153 participants, Figures 4 and 5, Table 2, supplementary data).

**Figure 6:**
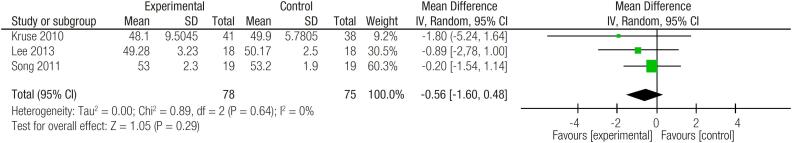
Meta-analysis of the balance improvement by Berg Balance Scale.

While observing the improvement in balance, as measured with the BBS and TUG, the meta-analyses of both tests did not show significant differences between the groups (MD 0.56 95% CI −1.60 to 0.48, 3 trials, 153 participants, low-certainty evidence; MD −0.63 95% CI −1.73 to 0.47, 4 trials, 185 participants, very low-certainty evidence, respectively; Figure 6 to 8 of the supplementary data).

Investigation of the publication bias was not possible owing to the small number of studies included (<10) (33).

### Results of outcomes that could not be plotted in the meta-analysis

Only Kruse and cols. evaluated the risk of falls (17), with a non-significance difference, in a follow-up of 12 months, 24% and 22% of the participants in the intervention and the control groups, respectively, fell once, while 17% and 18% of the participants in the intervention and the control groups, respectively, fell two or more times (RR 0.93, 95% CI 0.41 to 2.09; RR 0.93, 95% CI 0.36 to 2.40, respectively, 1 trial, 79 participants, low-certainty evidence, Table 2, supplementary data). Grewal and cols. evaluated the quality of life using the short-form health survey (SF-12) that includes a physical and a mental component score. At follow-up, the SF-12 did not reveal a significant difference between the groups; however, there was a mean difference of 12.78 in the mental score, in favor of the intervention (95% CI: 1.08 to 24.48, 1 trial, 35 participants, low-certainty evidence) (31).

For the secondary outcomes, Sartor and cols. evaluated the foot and ankle muscle function and the ABC Scale (30). After 12 weeks of follow-up, there was a difference between the groups in muscle function that favored the intervention group. In the foot physical examination and the ABC scores, there was no significant difference between the groups for any of the assessments. Two trials evaluated DPN progression: Sartor and cols. used the Michigan Neuropathy Screening Instrument (MNSI questionnaire and foot physical assessment) and Dixit and cols. used the Evaluation of Michigan Diabetic Neuropathy Score. There was no significant difference between the groups in the first trial but there was a significant difference in favor of the intervention in the second one (28,30). Only one study evaluated diabetic control, and using the glycated hemoglobin level (HbA1c), after 6 weeks of follow up there was no significant difference between the groups (Mean 7.0% [±1.01] and 6.94% [±1.12] in the intervention and control groups, respectively) (29). No trial reported the anthropometric data and adverse events of the patients studied.

## DISCUSSION

We hypothesized that structured exercise programs for DPN patients would promote balance improvement, which would lead to a lower risk of falls and a decrease in the fear of falling. Thus, we performed this systematic review and meta-analysis. Eight trials fulfilled our eligibility criteria and were included in this review. Four hundred and fifty-seven individuals with DPN were randomized to an exercise program for improving balance and strength or to no exercise program. Despite achieving significant results in favor of the intervention for balance and fear of falling, there was no difference between groups in the risk of falls. Additionally, the 95% CIs for these outcomes were very wide, resulting in a low quality of evidence according to the GRADE approach.

Many trials have evaluated the efficacy of exercise programs to prevent falls in older patients, and the Prevention of Falls Network Europe developed a taxonomy that classifies exercise modality as follows: (1) gait, balance, and functional training; (2) strength/resistance training; (3) flexibility; (4) three-dimensional (3D) exercise (Tai Chi, Qigong, dance), (5) general physical activity, (6) endurance, and (7) others (34,35). A systematic review performed by the Cochrane collaboration assessed the effect of these exercise interventions in preventing falls in community-dwelling older patients (35). With a high certainty of evidence, the meta-analysis showed that exercise reduces the incidence of falls by 23% (RR 0.77, 95% CI 0.71 to 0.83, 12,981 participants, 59 studies). The most effective exercise modality in reducing falls includes the balance and functional exercises, followed by different combinations of these modalities (typically balance and functional exercises plus resistance exercises) and Tai Chi. They were uncertain of the effect of the resistance exercises (without balance and functional exercises), dance, and walking (35). Taking into account that gait, balance and functional training involve, respectively, correction of the walking technique, as well as level and direction, transference of body weight from one part of the body to another, training stimulus, the three studies included in our review that assessed the risk of falls used an intervention according to this category (17,18,31). Although the participants in our review were different from those in Cochrane's review (patients with DPN vs. the general population), the lack of significant differences between the intervention and control groups in the risk of falls in our review can be owing to the small sample sizes and durations of follow-up in these studies. This does not necessarily reflect the ineffectiveness of the exercise programs.

Some studies have reported an improvement in the quality of life in diabetes patients who exercise (36,37). The only study that evaluated this outcome in our review used the SF-12 questionnaire but showed no difference between the groups; however, there was a significant difference in favor of the exercise for the mental score (31). An RCT using the SF-36 questionnaire showed that adults with type 2 diabetes mellitus are likely to benefit from adopting an exercise training regimen regardless of the exercise training modality (aerobic, resistance, or a combination of both); however, combined aerobic/resistance exercises produced greater benefits in some SF-36 domains (e.g. mental health) (38). Physical activity interventions have also shown improvement in the glycemic control (39,40). In our review, only one study evaluated this outcome but was unable to demonstrate a significant difference between the two groups owing to an insufficient follow-up duration.

Falls are one of the major concerns for older people with diabetes mellitus, and they may not be attributed solely to DPN (41). In a population-based study, the incidence of falls in a group of older patients with diabetes was 39%. Falls occurred more frequently in women, patients with poor diabetic control, patients requiring assistance with mobility, and those who had a history of stroke (42). A systematic review of diabetes mellitus and the risk of falls showed that the older adults with diabetes mellitus are at a greater risk of falls. The risk of falls seemed more pronounced among both genders rather than in women only, and this association was more pronounced in insulin-treated patients. In our review, there was no sex-based difference between groups, and only two studies mentioned the proportion of patients taking insulin (14,28).

While this review was being performed, an unregistered systematic review was published on the same subject. Gu and Dennis compared the improvement in balance with respect to the lower limb strengthening exercises, walking programs, and Tai Chi with other exercise programs for fall prevention in type 2 diabetes and DPN patients (43). The authors concluded that there is insufficient long-term follow-up data to determine whether the improvements in balance or strength resulted in a decrease in the risk of falls in the community setting (43). Although the risk of falls is quite similar to the results shown here, there are some differences between this review and ours. First, they included study designs other than RCTs. Second, they included studies with type 2 diabetics only; therefore, they did not include four studies that were included in our review (14,17,28,29). Third, they did not perform a meta-analysis for the improvement in balance and the fear of falling. Fourth, they included those studies in which the control group also received an exercise program, some of which were meant for improving balance and strength (25,26,44). As our objective was not to compare the efficacy of two kind of exercise programs, we only included trials whose control was compound for non-intervention.

Our systematic review has limitations, with the main one related to the small number of trials and patients included. The studies were single-center trials that tend to provide larger treatment effects than multicenter RCT; hence, the results of these studies should be carefully used in decision making (45). Additionally, no trial reported any adverse events as outcomes, and only one study presented the outcomes in a sufficient long-term follow-up period (12 months). Regarding balance performance, it is important to note that the improvement was found for only one type of static balance test. The methodological quality of the included studies was also an important limitation, since most studies had an unclear risk of detection and selection biases. The low quality of evidence according to the GRADE approach for the primary outcomes means that future studies, especially RCTs with large sample sizes and a proper methodology may still yield different results.

## CONCLUSION

### Implications for practice

Our review showed a significant difference between the two groups that favored the intervention for the improvement of balance and the fear of falling. No significant difference in the risk of falls was observed between the groups. However, as the literature with high quality of evidence shows that exercise reduces falls in older patients living in the community, this lack of significant difference between the groups may be owing to the small sample size and the duration of follow-up and not necessarily due to the ineffectiveness of the intervention.

### Implications for research

Randomized clinical trials with large sample sizes and proper methodology are needed to evaluate the real effect of the exercise programs on the risk of falls in DPN patients.

## Figures and Tables

**Figure 7:**
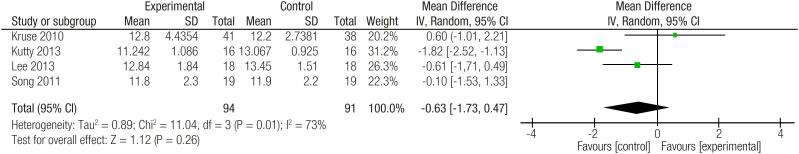
Meta-analysis of the balance improvement by Timed Up and Go.

**Figure 8:**
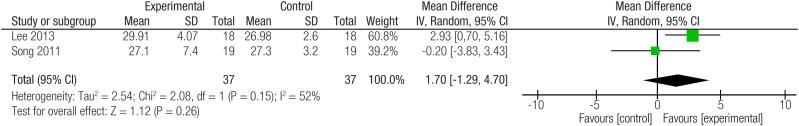
Meta-analysis of the balance improvement by Functional Reach Test.

## Supplementary Data

**Table 2:** Summary of findings Efficacy of Exercise on Balance, Fear of Falling, and risk of Falls in Patients with Diabetic Peripheral Neuropathy: A Systematic Review and Meta-Analysis Patient or population: Patients with Diabetic Polyneuropathy Intervention: Exercise programs Comparison: No Exercises

Outcomes	Anticipated absolute effects* (95% CI)		Relative effect (95% CI	Nº of participants (studies)	Certainty of the evidence (GRADE)	Comments
	Risk with placebo	Risk with Teste de Equilibrio (parado numa perna)				
One-leg stance test with eyes open	The mean one-leg stance test with eyes open was 0	MD **3.7 higher** (0.64 higher to 6.76 higher)	–	153 (3 RCTs)	⊗ ⊗ 〇〇 LOW^a^,^b^	The intervention may improve one-leg stance test with eyes open.
Fall Efficacy Scale (Fear of Falling)	The mean fall Efficacy Scale was 0	MD 2.42 lower (4.7 lower to 0.15 lower)	–	185 (3 RCTs)	⊗⊗ 〇〇 LOW^b^,^c^	Intervention may reduce fear of falling.
One-leg stance test with eyes closed	The mean one-leg stance test with eyes closed was 0	MD 1.07 higher (0.34 higher to 1.79 higher)	–	153 (3 RCTs)	⊗⊗ 〇〇 LOW^a^,^b^	The intervention may improve one-leg stance test with eyes closed.
Risk of Falls (once in 12 months follow-up)	184 per 1.000	171 per 1.000 (66 to 442)	RR 0.93 (0.36 to 2.40)	79 (1 RCT)	⊗⊗ 〇〇 LOW^b^,^d^	Due to very serious imprecision, there is no clear effect of intervention on the risk of falls.

* The risk in the intervention group (and its 95% confidence interval) is based on the assumed risk in the comparison group and the relative effect of the intervention (and its 95% CI).

CI: Confidence interval; MD: Mean difference; RR: Risk ratio

**Grading of Recommendations Assessment, Development, and Evaluation (GRADE) Working Group grades of evidence** **High certainty:** We are very confident that the true effect lies close to that of the estimate of the effect **Moderate certainty:** We are moderately confident in the effect estimate: The true effect is likely to be close to the estimate of the effect, but there is a possibility that it is substantially different **Low certainty:** Our confidence in the effect estimate is limited: The true effect may be substantially different from the estimate of the effect **Very low certainty:** We have very little confidence in the effect estimate: The true effect is likely to be substantially different from the estimate of effect

Explanations

a. Unclear selection bias risk in most studies included

b. No achievement of optimal information size

c. Unclear risk of detection bias

d. Wide confidence interval

### Search Strategy: 31/Dec/2019: PUBMED

# 1 “Diabetic Neuropathies”[Mesh] OR (Diabetic Neuropathy) OR (Neuropathies, Diabetic) OR (Neuropathy, Diabetic) OR (Diabetic Polyneuropathy) OR (Diabetic Polyneuropathies) OR (Polyneuropathies, Diabetic) OR (Polyneuropathy, Diabetic) OR (Asymmetric Diabetic Proximal Motor Neuropathy) OR (Diabetic Asymmetric Polyneuropathy) OR (Asymmetric Polyneuropathies, Diabetic) OR (Asymmetric Polyneuropathy, Diabetic) OR (Diabetic Asymmetric Polyneuropathies) OR (Polyneuropathies, Diabetic Asymmetric) OR (Polyneuropathy, Diabetic Asymmetric) OR (Diabetic Autonomic Neuropathy) OR (Autonomic Neuropathies, Diabetic) OR (Autonomic Neuropathy, Diabetic) OR (Diabetic Autonomic Neuropathies) OR (Neuropathies, Diabetic Autonomic) OR (Neuropathy, Diabetic Autonomic) OR (Symmetric Diabetic Proximal Motor Neuropathy) OR (Diabetic Amyotrophy) OR (Amyotrophies, Diabetic) OR (Amyotrophy, Diabetic) OR (Diabetic Amyotrophies) OR (Diabetic Neuralgia) OR (Diabetic Neuralgias) OR (Neuralgias, Diabetic) OR (Diabetic Neuropathy, Painful) OR (Diabetic Neuropathies, Painful) OR (Neuropathies, Painful Diabetic) OR (Neuropathy, Painful Diabetic) OR (Painful Diabetic Neuropathies) OR (Painful Diabetic Neuropathy) OR (Neuralgia, Diabetic) OR (Diabetic Mononeuropathy) OR (Diabetic Mononeuropathies) OR (Mononeuropathies, Diabetic) OR (Mononeuropathy, Diabetic) OR (Diabetic Mononeuropathy Simplex) OR (Diabetic Mononeuropathy Simplice) OR (Mononeuropathy Simplex, Diabetic) OR (Mononeuropathy Simplice, Diabetic) OR (Simplex, Diabetic Mononeuropathy) OR (Simplice, Diabetic Mononeuropathy) OR (Peripheral Diabetic Neuropathy) OR (Peripheral Diabetic Neuropathies) OR (Diabetic Peripheral Neuropathy) OR (Diabetic Peripheral Neuropathies) OR (Proximal Diabetic Neuropathy) OR (Proximal Diabetic Neuropathy) OR “Diabetes Complications”[Mesh] OR (Diabetes Complications) OR (Diabetes-Related Complications) OR (Diabetes Related Complications) OR (Diabetes-Related Complication) OR (Diabetic Complications) OR (Diabetic Complication) OR (Complications of Diabetes Mellitus) OR (Diabetes Mellitus Complication) OR (Diabetes Mellitus Complications) OR (Distal Symmetric Polyneuropathy) OR (Sensorimotor Distal Symmetric Polyneuropathy) OR (Diabetic Sensorimotor Polyneuropathy)

#2 “Exercise”[Mesh] OR (Exercises) OR (Exercise, Physical) OR (Exercises, Physical) OR (Physical Exercise) OR (Physical Exercises) OR (Exercise, Isometric) OR (Exercises, Isometric) OR (Isometric Exercises) OR (Isometric Exercise) OR (Exercise, Aerobic) OR (Aerobic Exercises) OR (Exercises, Aerobic) OR (Aerobic Exercise) OR “Warm-Up Exercise”[Mesh] OR (Exercise, Warm-Up) OR (Exercises, Warm-Up) OR (Warm Up Exercise) OR (Warm-Up Exercises) OR (Warmup Exercise) OR (Exercise, Warmup) OR (Exercises, Warmup) OR (Warmup Exercises) OR (Warming-Up Exercise) OR (Exercise, Warming-Up) OR (Exercises, Warming-Up) OR (Warming Up Exercise) OR (Warming-Up Exercises) OR “Exercise Movement Techniques”[Mesh] OR (Movement Techniques, Exercise) OR (Exercise Movement Techniques) OR (Pilates-Based Exercises) OR (Exercises, Pilates-Based) OR (Pilates Based Exercises) OR (Pilates Training) OR (Training, Pilates) OR “Exercise Therapy”[Mesh] OR (Therapy, Exercise) OR (Exercise Therapies) OR (Therapies, Exercise) OR “Resistance Training”[Mesh] OR (Training, Resistance) OR (Strength Training) OR (Training, Strength) OR (Weight-Lifting Strengthening Program) OR (Strengthening Program, Weight-Lifting) OR (Strengthening Programs, Weight-Lifting) OR (Weight Lifting Strengthening Program) OR (Weight-Lifting Strengthening Programs) OR (Weight-Lifting Exercise Program) OR (Exercise Program, Weight-Lifting) OR (Exercise Programs, Weight-Lifting) OR (Weight Lifting Exercise Program) OR (Weight-Lifting Exercise Programs) OR (Weight-Bearing Strengthening Program) OR (Strengthening Program, Weight-Bearing) OR (Strengthening Programs, Weight-Bearing) OR (Weight Bearing Strengthening Program) OR (Weight-Bearing Strengthening Programs) OR (Weight-Bearing Exercise Program) OR (Exercise Program, Weight-Bearing) OR (Exercise Programs, Weight-Bearing) OR (Weight Bearing Exercise Program) OR (Weight-Bearing Exercise Programs) OR “Muscle Stretching Exercises”[Mesh] OR (Exercise, Muscle Stretching) OR (Exercises, Muscle Stretching) OR (Muscle Stretching Exercise) OR (Dynamic Stretching) OR (Stretching, Dynamic) OR (Isometric Stretching) OR (Stretching, Isometric) OR (Active Stretching) OR (Stretching, Active) OR (Static-Active Stretching) OR (Static Active Stretching) OR (Stretching, Static-Active) OR (Static Stretching) OR (Stretching, Static) OR (Passive Stretching) OR (Stretching, Passive) OR (Relaxed Stretching) OR (Stretching, Relaxed) OR (Static-Passive Stretching) OR (Static Passive Stretching) OR (Stretching, Static-Passive) OR (Ballistic Stretching) OR (Stretching, Ballistic) OR (Proprioceptive Neuromuscular Facilitation (PNF) Stretching) OR “Physical Conditioning, Human”[Mesh] OR (Conditioning, Human Physical) OR (Conditionings, Human Physical) OR (Human Physical Conditioning) OR (Human Physical Conditionings) OR (Physical Conditionings, Human) OR “Swimming”[Mesh] OR “Walking”[Mesh] OR (Ambulation) OR “Motor Activity”[Mesh] OR (Activities, Motor) OR (Activity, Motor) OR (Motor Activities) OR (Physical Activity) OR (Activities, Physical) OR (Activity, Physical) OR (Physical Activities) OR (Locomotor Activity) OR (Activities, Locomotor) OR (Activity, Locomotor) OR (Locomotor Activities) OR “Gymnastics”[Mesh] OR (Gymnastic) OR (Calisthenics) OR (Calisthenic) OR “Physical Education and Training”[Mesh] OR (Physical Education, Training) OR (Physical Education) OR (Education, Physical) OR “Physical Therapy Modalities”[Mesh] OR (Modalities, Physical Therapy) OR (Modality, Physical Therapy) OR (Physical Therapy Modality) OR (Physiotherapy (Techniques)) OR (Physiotherapies (Techniques)) OR (Physical Therapy Techniques) OR (Physical Therapy Technique) OR (Techniques, Physical Therapy) OR “Physical Fitness”[Mesh] OR (Fitness, Physical) OR “Physical Therapy Specialty”[Mesh] 0R (Specialty, Physical Therapy) OR (Therapy Specialty, Physical) OR (Physiotherapy Specialty) OR (Specialty, Physiotherapy)

#3 “Exercise”[Mesh] OR (Exercises) OR (Exercise, Physical) OR (Exercises, Physical) OR (Physical Exercise) OR (Physical Exercises) OR (Exercise, Isometric) OR (Exercises, Isometric) OR (Isometric Exercises) OR (Isometric Exercise) OR (Exercise, Aerobic) OR (Aerobic Exercises) OR (Exercises, Aerobic) OR (Aerobic Exercise) OR (Warm-Up Exercise) OR (Exercise, Warm-Up) OR (Exercises, Warm-up) OR (Warm Up Exercise) OR (Warm Up Exercises) OR “Motor Activity”[Mesh] OR (Activities, Motor) OR (Activity, Motor) OR (Motor Activities) OR (Physical Activity) OR (Activities, Physical) OR (Activity, Physical) OR (Physical Activities) OR (Locomotor Activity) OR (Activities, Locomotor) OR (Activity, Locomotor) OR (Locomotor Activities) OR “Exercise Therapy”[Mesh] OR (Therapy, Exercise) OR (Exercise Therapies) OR (Therapies, Exercise) OR “Exercise Movement Techniques”[Mesh] OR (Movement Techniques, Exercise) OR (Exercise Movement Techniques) OR “Resistance Training”[Mesh] OR (Training, Resistance) OR (Strength Training) OR (Training, Strength) OR (Weight-Lifting Strengthening Program) OR (Strengthening Program, Weight-Lifting) OR (Strengthening Programs, Weight-Lifting) OR (Weight Lifting Strengthening Program) OR (Weight-Lifting Strengthening Programs) OR (Weight-Lifting Exercise Program) OR (Exercise Program, Weight-Lifting) OR (Exercise Programs, Weight-Lifting) OR (Weight Lifting Exercise Program) OR (Weight-Lifting Exercise Programs) OR (Weight-Bearing Strengthening Program) OR (Strengthening Program, Weight-Bearing) OR (Strengthening Programs, Weight-Bearing) OR (Weight Bearing Strengthening Program) OR (Weight-Bearing Strengthening Programs) OR (Weight-Bearing Exercise Program) OR (Exercise Program, Weight-Bearing) OR (Exercise Programs, Weight-Bearing) OR (Weight Bearing Exercise Program) OR (Weight-Bearing Exercise Programs) OR “Physical Fitness”[Mesh] OR (Fitness, Physical) OR (Physical Conditioning, Human) OR (Conditioning, Human Physical) OR (Conditionings, Human Physical) OR (Human Physical Conditioning) OR (Human Physical Conditionings) OR (Physical Conditionings, Human) OR “Physical Therapy Modalities”[Mesh] OR (Modalities, Physical Therapy) OR (Modality, Physical Therapy) OR (Physical Therapy Modality) OR (Physiotherapy (Techniques)) OR (Physiotherapies (Techniques)) OR (Physical Therapy Techniques) OR (Physical Therapy Technique) OR (Techniques, Physical Therapy) OR “Physical Therapy Specialty”[Mesh] OR (Specialty, Physical Therapy) OR (Therapy Specialty, Physical) OR (Physiotherapy Specialty) OR (Specialty, Physiotherapy) OR “Muscle Stretching Exercises”[Mesh] OR (Exercise, Muscle Stretching) OR (Exercises, Muscle Stretching) OR (Muscle Stretching Exercise) OR (Dynamic Stretching) OR (Stretching, Dynamic) OR (Isometric Stretching) OR (Stretching, Isometric) OR (Active Stretching) OR (Stretching, Active) OR (Static-Active Stretching) OR (Static Active Stretching) OR (Stretching, Static-Active) OR (Static Stretching) OR (Stretching, Static) OR (Passive Stretching) OR (Stretching, Passive) OR (Relaxed Stretching) OR (Stretching, Relaxed) OR (Static-Passive Stretching) OR (Static Passive Stretching) OR (Stretching, Static-Passive) OR (Ballistic Stretching) OR (Stretching, Ballistic) OR (Proprioceptive Neuromuscular Facilitation (PNF) Stretching) OR “Physical Conditioning, Human”[Mesh] OR (Conditioning, Human Physical) OR (Conditionings, Human Physical) OR (Human Physical Conditioning) OR (Human Physical Conditionings) OR (Physical Conditionings, Human) OR “Swimming”[Mesh] OR “Walking”[Mesh] OR (Ambulation) OR “Gymnastics”[Mesh] OR (Gymnastic) OR (Calisthenics) OR (Calisthenic) OR “Hydrotherapy”[Mesh] OR (Hydrotherapies) OR (Whirlpool Baths) OR (Bath, Whirlpool) OR (Baths, Whirlpool) OR (Whirlpool Bath)

**#4 (randomized controlled trial[pt] OR controlled clinical trial[pt] OR randomized[tiab] OR placebo[tiab] OR drug therapy[sh] OR randomly[tiab] OR trial[tiab] OR groups[tiab] NOT (animals [mh] NOT humans [mh]))***

*Cochrane filter for randomized clinical trials

#1 AND #2 OR #3 AND #4, FILTRO HUMANOS, 1482

### EMBASE

#1 ‘diabetic neuropathy’/exp OR ‘diabetes neuropathy’ OR ‘diabetic neuritis’ OR ‘diabetic neuropathies’ OR ‘diabetic polyneuritis’ OR ‘diabetic polyneuropathy’ OR ‘neurpathy, diabetic’

#2 ‘aerobic exercise’/exp OR ‘aerobic dance’ OR ‘aerobic dancing’ OR ‘aerobics’ OR ‘aerobics exercise’ OR ‘dancing, aerobic’ OR ‘exercise, aerobic’ OR ‘low impact aerobic exercise’ OR ‘low impact aerobics’ OR ‘step aerobics’ OR ‘anaerobic exercise’/exp OR ‘anaerobic exercise work’ OR ‘anaerobic work’ OR ‘ aquatic exercise’/exp OR ‘execise, aquatic’ OR ‘ exercise’/exp OR ‘biometric exercise’ OR ‘effort’ OR ‘exercise capacity’ OR ‘exercise performance’ OR ‘exercise training’ OR ‘exertion’ OR ‘fitness training’ OR ‘physical conditioning, human’ OR ‘physical effort’ OR ‘physical exercise’ OR ‘physical exertion’ OR ‘restraint, physical’ OR ‘dynamics exercise’/exp OR ‘exercise, dynamic’ OR ‘endurance training’/exp OR ‘endurance exercise’ OR ‘endurance exercise training’ OR ‘exercise intensity’/exp OR ‘kinesiotherapy’/exp OR ‘exercise movement techniques’ OR ‘exercise therapy’ OR ‘exercise treatment’ OR ‘kinesitherapy’ OR ‘therapeutic exercise’ OR ‘therapy exercise’ OR ‘treatment, exercise’ OR ‘isometric exercise’/exp OR ‘exercise, isometric’ OR ‘isometric endurance’ OR ‘isometric endurance test’ OR ‘isometric training’ OR ‘isotonic exercise’/exp OR ‘static exercise’/exp OR ‘exercise, static’ OR ‘stretching’ ‘exercise’/exp OR ‘muscle stretching exercises’ OR ‘stretching exercises’ OR ‘pilates’/exp OR ‘pilates exercise’ OR ‘resistance training’/exp OR ‘resistance exercise’ OR ‘resistance exercise training’ OR ‘strength training’ OR ‘weight bearing exercise’ OR ‘treadmill exercise’/exp OR ‘exercise, treadmill’ OR ‘treadmill running’ OR ‘warm up’/exp OR ‘warm-up exercise’ OR ‘warming up exercise’ OR ‘warmup’ OR ‘muscle exercise’/exp OR ‘muscle endurance’ OR ‘muscle exertion’ OR ‘muscular exercise’ OR ‘muscular exertion’

#1 AND #2 = 209

### LILACS, Enfermagem, IBECS VIA PORTAL BVS EM FORMULÁRIO IAHX

#1 MH:” Neuropatias Diabéticas” OR (Neuropatías Diabéticas) OR (Diabetic Neuropathies) OR (Acropatia Diabética Ulceromutilante) OR (Amiotrofia Diabética) OR (Neuropatia Autônoma Diabética) OR (Nevralgia Diabética) OR (Polineuropatia Diabética) OR MH:C10.668.829.300$ OR MH:C19.246.099.937$ OR MH:”Complicações do Diabetes” OR (Complicaciones de la Diabetes) OR (Diabetes Complications) OR (Complicações da Diabetes) OR (Complicações Diabéticas) OR MH: C19.246.099$

#2 MH:”Exercício” OR (Ejercicio) OR (Exercise) OR (Exercício Aeróbico) OR (Exercício Isométrico) OR (Exercício Físico) OR MH:”Atividade Motora” OR (Actividad Motora) OR (Motor Activity) OR (Atividade Locomotora) OR (Atividade Física) OR MH:”Terapia por Exercício” OR (Terapia por Ejercicio) OR (Exercise Therapy) OR MH:”Técnicas de Exercício e de Movimento” OR (Técnicas de Ejercicio con Movimientos) OR (Exercise Movement Techniques) OR (Técnicas de Movimentos do Exercício) OR MH:”Exercícios de Alongamento Muscular” OR (Ejercicios de Estiramiento Muscular) OR (Muscle Stretching Exercises) OR (Exercícios de Estiramento Muscular) OR (Exercício de Alongamento Muscular) OR MH:G11.427.590.530.698.277$ OR MH:I03.350$ OR MH:F01.145.632$ OR MH:G11.427.590.530.698$ OR MH:E02.779.483$ OR MH:E02.831.387$ OR MH:E02.779.474$ OR MH:E02.779.483.750$ OR MH:E02.831.387.750$ OR MH:G11.427.590.530.698.277.249$ OR MH:I03.350.249$

Total= 147

COCHRANE

Search Name:

Date Run: 01/01/2020 03:11:54

Comment:[Table t3]

**Table t3:**

ID	Search	Hits
#13	MeSH descriptor: [Diabetic Neuropathies] explode all trees	1869
#14	MeSH descriptor: [Exercise] explode all trees	22949
#15	Physical Fitness	8334
#16	MeSH descriptor: [Exercise] explode all trees	22949
#17	#14 OR #15 OR #16	28493
#18	#13 AND #17	43
